# Characterization of aspirin-sensitivity in patients with asthma and chronic rhinosinusitis without a history of symptoms upon acetyl-salicylic acid (ASA) ingestion: a case series

**DOI:** 10.1186/1939-4551-8-S1-A199

**Published:** 2015-04-08

**Authors:** Phelipe Souza, Lucas Brom, Thais Nociti, Janaina M Melo, Karine Boufleur, Priscila Palhas, Daniel Cordeiro, Ullissis Menezes, Edwin Tanashiro, Wilma Lima, L Karla Arruda

**Affiliations:** 1 Clinical Hospital of Ribeirão Preto Medical School, Brazil; 2 Ribeirao Preto Medical School, University of Sao Paulo, Brazil; 3 Brazilian Association of Allergy and Immunology, Brazil

## Background

Sensitivity to Non-steroidal Anti-inflammatory Drugs (NSAIDs) is a possible cause of exacerbations in patients with asthma. NSAID sensitivity associated to chronic rhinosinusitis with nasal polyps features Aspirin-Exacerbated Respiratory Disease (AERD). Patients with AERD generally require long-term treatment with inhaled corticosteroids for moderate to severe persistent asthma. Respiratory reactions may begin within minutes to hours after ingestion of NSAIDs.

## Methods

Descriptive study of a case series of five patients with asthma, chronic rhinosinusitis with nasal polyps, without a history of symptoms upon NSAIDs ingestion, who underwent single-blind, placebo controlled, Oral Challenge Test (OCT) with Acetylsalicylic Acid (ASA). OCTs were performed according to the recommendations of the European Network for Drug Allergy (ENDA), with ASA cumulative dose of 500mg, starting at 50mg, with subsequent doses of 100mg, 150mg, 200mg, administered at 15 minute intervals, to investigate sensitivity to ASA. All patients signed an informed consent.

## Results

Mean age of the five patients was 55 years, ranging from 42 to 62 years. Three patients were female. No previous use of ASA was reported by the patients, and two of them denied using any other NSAIDs. Three patients had a history of Diclofenac intake, and one of Nimesulide intake, without perceived reactions. Four patients had positive skin tests to inhalant allergens. Three patients presented positive OCTs with ASA, with the following reactions: two patients presented cough, wheezing and dyspnea with 500mg cumulative doses; one patient progressed to anaphylaxis after a cumulative dose of 300mg, presenting wheezing, dyspnea, tachycardia, dizziness, 33% drop on Peak Flow measurement and fall of oxygen saturation to 89%.

## Conclusions

Despite absence of a consistent history of symptoms upon ingestion of ASA and/or other NSAIDs, three out of five patients in our series of patients with asthma, chronic rhinosinusitis and nasal polyps presented positive OCTs with ASA, comprising mostly respiratory symptoms. Our data reinforces the importance of OCTs with ASA as an effective method for characterizing ASA/NSAIDs sensitive asthmatic patients. A positive OCT may indicate desensitization as a complementary treatment, which is well established as a successful method for reducing recurrences of nasal polyps, improving smell and asthma control.

